# Evaluation of the rate, pattern and appropriateness of antibiotic prescription in a cohort of pilgrims suffering from upper respiratory tract infection during the 2018 Hajj season

**DOI:** 10.1099/acmi.0.000338

**Published:** 2022-04-25

**Authors:** Hamid Bokhary, Hajj Research Team, Osamah Barasheed, Hala B. Othman, Burhanudin Saha, Harunor Rashid, Grant A. Hill-Cawthorne, Moataz Abd El Ghany

**Affiliations:** ^1^​ School of Public Health, The University of Sydney, Sydney, NSW, Australia; ^2^​ The Westmead Institute for Medical Research, Westmead, NSW, Australia; ^3^​ Sydney Institute for Infectious Diseases, The University of Sydney, Westmead, NSW, Australia; ^4^​ University Medical Centre, Umm Al-Qura University, Makkah, Saudi Arabia; ^5^​ King Abdullah Medical City, Makkah, Saudi Arabia; ^6^​ Clinical Pathology Department, Faculty of Medicine, Ain-Shams University, Cairo, Egypt; ^7^​ National Centre for Immunization Research and Surveillance of Vaccine Preventable Diseases, Kids Research Institute, The Children’s Hospital at Westmead, Westmead, NSW, Australia

**Keywords:** Hajj, antibiotic, prescription, appropriateness, upper respiratory tract infection

## Abstract

Hajj is associated with an increased risk of the transmission of infectious diseases including upper respiratory tract infections (URTIs). It can be a focal point for the emergence, persistence and dissemination of antimicrobial-resistant (AMR) bacteria. The overuse of antibiotics during Hajj can promote the development of antimicrobial resistance. Little information is known regarding the true appropriateness of prescribing antibiotics for treating URTIs during Hajj. Here we studied the rate, patterns and appropriateness of antibiotic prescription among a cohort of pilgrims who were treated for URTIs during the 2018 Hajj season. Adult pilgrims who sought medical services for URTIs [presenting with coryza, runny nose, nasal irritation, nasal congestion, cough, sore throat, headache or fever (even if subjective)] within the Holy sites were enrolled in this study and consented to provide swabs and medical information. A total of 121 pilgrims were enrolled, with the majority (60.3 %) originating from North African Arab countries. Most were male (89.3 %) with a median age of 45 years. Bacterial infections were detected in 7.3 % (*n*=9) of the URTI cases. The identified bacteria included *Haemophilus influenzae* (*n*=6, all resistant to ampicillin), *Streptococcus pneumoniae* (*n*=2), *Staphylococcus aureus* (*n*=1, resistant to oxacillin) and *Moraxella catarrhalis* (*n*=1, resistant to ampicillin and trimethoprim/sulfamethoxazole). The antibiotic prescription rate was 52.1%, most of which was amoxicillin (81 %). The data demonstrated that the proportion of appropriate practices in treating bacterial URTIs in this cohort was 45.5 %. This study highlights the need for implementing laboratory identification of the aetiological agents and related AMR profiles when treating URTIs in Hajj, rather than relying on clinical assessment alone.

## Data Summary

The authors confirm all supporting data, code and protocols have been provided within the article or through supplementary data files.

## Introduction

Hajj is an annual diverse religious mass gathering that attracts more than two million Muslim pilgrims from over 180 countries around the globe [1]. This enormously diverse population of pilgrims and the nature of the activities performed (e.g. crowded accommodation, prolonged stay in tents) can promote the dissemination of infectious diseases [2]. Hajj has been associated with the emergence and dissemination of airborne (e.g. meningitis), foodborne (e.g. diarrhoea), bloodborne (e.g. viral hepatitis) and zoonotic infectious diseases within the host country and globally [2]. Despite substantial advances in food and water hygiene, vaccination policy [3], and healthcare services available during Hajj through the local authorities, Hajj still represents an ideal environment for the transmission of infectious diseases and, subsequently, antimicrobial-resistant (AMR) pathogens [2, 4].

The threat of AMR is increasing globally [5]. A recent report of the World Health Organization’s (WHO) Global Antimicrobial Surveillance System (GLASS) has demonstrated high levels of resistance to many bacterial species associated with serious and common infections, including *Klebsiella pneumoniae *, *Staphylococcus aureus* and *Streptococcus pneumoniae *. Currently global resistance-associated mortality accounts for 70 000 deaths a year, and it is estimated to top 10 million people per year in 2050 if infectious disease levels remain as today’s [6]. It has been estimated that this may cost up to US$300 million due to associated premature death and up to a US$100 trillion loss from the global economy [6].

The challenges of AMR are complex and multifaceted, with AMR drivers persisting and interlinked between different ecologies including humans, animals, plants, food and the environment [7]. The main drivers of AMR include lack of access to clean drinking water, sanitation and hygiene for both humans and animals, poor disease prevention and infection control on healthcare facilities, poor access to vaccines and diagnostics, lack of awareness and knowledge, and lack of enforcement of legislation. A number of studies have highlighted the role of human activities including overuse and misuse of antibiotics, international travel [8] and forced displacement in promoting [9, 10] the dissemination of multi-drug-resistant (MDR) bacteria globally.

Recently, a number of studies have highlighted Hajj as a focal point for the emergence and dissemination of AMR globally [4, 11]. Multiple factors, including the demographics of Hajj pilgrims and sanitation infrastructure, can potentially favour AMR dissemination [12]. The dissemination of AMR bacteria in Hajj settings is multifactorial, with environmental, enteric and respiratory origins. An example of environmental causes is the role of wastewater in the transmission of MDR bacteria causing waterborne diseases, most of which in Hajj are enteric bacteria [7]. During Hajj, bacteria of enteric origins causing food-borne diseases are well documented to harbour AMR genes, such as *bla*_CTX-M-15_ and *bla*_NDM_, and contribute to the emergence of AMR bacteria within Hajj [13, 14]. These organisms may also circulate within the environment if not properly treated, promoting further persistence of AMR bacteria locally [7]. Although these origins for AMR organisms are well addressed in the literature, the acquisition of AMR within bacteria of respiratory origin lacks similar depth.

Respiratory infectious illnesses are the most studied communicable diseases associated with Hajj. Air-borne diseases in Hajj are caused by viruses, bacteria or both [15]. Respiratory viruses include influenza viruses A and B, parainfluenza viruses, respiratory syncytial virus and adenovirus. Common respiratory bacteria include *Haemophilus influenzae *, *Klebsiella pneumoniae *, *Streptococcus pneumoniae* and *Staphylococcus aureus* [15]. Some studies have examined AMR bacteria that cause respiratory tract infections and the probability of developing MDR bacteria in Hajj [16, 17].

Upper respiratory tract infections (URTIs) and their associated symptoms are highly common health complaints during Hajj [18, 19]. More than half of pilgrims have a URTI symptom whilst undertaking their Hajj journey [20]. The most common organisms found to be associated with respiratory infections during Hajj are bacterial: *H. influenzae* and *Streptococcus pneumoniae* [15, 19]. However, most of the reported URTIs in Hajj are likely to be of viral aetiology, and the most common causes following testing are human rhinoviruses [21–23]. Conversely, up to 99 % of pilgrims may receive antibiotics for respiratory complaints during Hajj [21, 24], with only 40 % of these prescriptions being appropriate [24]. This is of considerable importance, as the inappropriate use of antibiotics may accelerate the rate of AMR emergence during the event [6, 25]. The most commonly prescribed antibiotic in Hajj is amoxicillin, often combined with clavulanic acid [26], to which most of the AMR organisms documented during the pilgrimage are resistant to [27]. A corresponding lack of AMR profiling for Hajj-associated URTI organisms means that there is limited knowledge on the effectiveness of such prescription practices. Therefore, the patterns of prescribing antibiotics and their appropriateness for URTIs during Hajj need to be further investigated.

A number of studies have evaluated the rate of antibiotic use among pilgrims from different origins. There was a reported 61.8, 53.8 and ~48 % antibiotic use in pilgrims from Malaysia, France and India, respectively [16, 24, 28]. A recent study on Hajj-deployed physicians reported that the decision to prescribe antibiotics for URTIs is not affected by the impact of Hajj on healthcare services [29]. Tools such as guidelines and rapid test kits are recommended when tackling AMR [25, 29], but so far none are used during Hajj.

This study explores the features of a cross-sectional sample of pilgrims complaining of URTI symptoms that sought medical care during the 2018 Hajj season, to determine the bacterial causative agents associated with URTIs, determine their AMR profile and link these to the antibiotics that were prescribed to them.

## Methods

### Study population and design

Adult pilgrims with URTI symptoms seeking medical services within Ministry of Health (MoH) facilities during the ritual were approached for potential recruitment. Trained investigators who received mandatory Hajj vaccines were distributed to various health facilities in Mina and Arafat. These are the areas where pilgrims are most likely to be present during the Hajj ritual. Pilgrims were invited if they exhibited or expressed any of the following symptoms or signs (inclusion criteria): coryza, runny nose, nasal irritation, nasal congestion, cough, sore throat, headache or fever (even if subjective). Consent was obtained for all those who agreed to participate in the study. Patient information (year of birth, gender and origin) were recorded including clinical data (brief URTI-related history and examination) and prescription information (what the pilgrims took from any pharmacy). Oropharyngeal swabs were taken from pilgrims and kept in appropriate transport media/preservative (Amies transport media). Once collected, the swabs were kept at 4 °C and transferred on wet ice or refrigerated gel packs to the laboratories at King Abdullah Medical City (KAMC), MoH Makkah branch, Makkah, Saudi Arabia, on the same date of collection (within 6–8 h depending upon geographical and traffic considerations), where the samples were immediately processed [30, 31].

### Identification of respiratory bacteria

Immediately upon arrival, samples were prepared and processed for culturing and sensitivity according to standard procedures applied in the diagnostic laboratory at KAMC. Each swab was mounted and cultured on four plates including two blood agar plates (aerobic and anaerobic), a chocolate agar plate and a MacConkey agar plate. The plates were incubated at 35 °C for 24–48 h. Five colonies from each bacterial morphology grown were picked for further characterization. Bacterial identification was performed using the automated VITEK 2 COMPACT system (bioMérieux) according to the manufacturer’s instructions. Barcoded detection cards and a fluorogenic methodology were used for the detection of 120 Gram-positive bacterial species including *Staphylococcus* and *Streptococcus* species (card GP), 149 fermenting and non-fermenting Gram-negative bacteria, including *Acinetobacter* species, *Moraxella* group and *Pseudomonas* species (card GN); and *Haemophilus* species (card NH) (Table S1, available in the online version of this article)

### AMR susceptibility testing

The AMR phenotype of the identified bacteria was determined using VITEK 2 COMPACT systems as per the manufacturer’s instructions. The turbidimetric method was used by this system to identify the susceptibility profiles of *Staphylococcus* species (AST-P576), *Streptococcus* species (AST-P580) and *Moraxella* group (AST-N291). The susceptibility profile for *Haemophilus* species was determined using a Kirby Baur Disc Diffusion method according to Clinical Laboratory Standard Institute (CLSI) interpretative criteria [32].

### Statistical analysis

Clinical data and laboratory results were entered into the electronic platform RedCap (Vanderbilt University, Nashville, TN, USA). Then the data were anonymized and exported to Excel 2016 (Microsoft Office 2016; Microsoft), checked and correlated. The sensitivity, specificity, positive predictive value (PPV), negative predictive value (NPV), odds ratio (OR) and confidence intervals (CIs) were calculated along with the bacterial characterization and the clinical findings [33, 34]. All statistical calculations were done through Excel.

## Results

### Characteristics of the study population

In total, 163 pilgrims were enrolled during the 2018 Hajj, most of whom (74.2 %, *n*=121) agreed to provide oropharyngeal swabs and stayed for clinical assessment. The study population had a median age of 45 years [interquartile range (IQR) 36–54 years]. The majority of the pilgrims enrolled were male (male/female ratio 8.3 : 1). Only one pilgrim was recruited from a hospital within Mina, five from Arafat primary healthcare centres, and the rest were from primary healthcare centres within Mina. The majority of pilgrims (60.3 %, *n*=73) originated from North African Arab countries, including Egypt (22.3 %, *n*=27), Sudan (14.9 %, *n*=18), Algeria (11.6 %, *n*=14), Morocco (9.1 %, *n*=11) and Libya (2.6 %, *n*=3). Pilgrims from Saudi Arabia, India and Pakistan accounted for 14.9 % (*n*=18), 7.4 % (*n*=9) and 6.6 % (*n*=8), respectively (Table 1).

**Table 1.** The clinical, demographic and swab findings for 121 pilgrims during the 2018 Hajj with symptoms of upper respiratory tract infections

Characteristic	Pilgrims		Bacterial infection	
	*n*=121	(%)	*n*=9	(%)
Gender				
Male	108	(89.3)	8	(88.9)
Female	13	(10.7)	1	(11.1)
**Age, years**				
0–29	17	(14.0)	1	(11.1)
30–49	58	(48.0)	2	(22.2)
50–79	46	(38.0)	6	(66.7)
**Country of origin**				
Egypt	27	(22.3)	1	(11.1)
Saudi Arabia	18	(14.9)	1	(11.1)
Sudan	18	(14.9)	2	(22.2)
Algeria	14	(11.6)	0	(0.0)
Morocco	11	(9.1)	2	(22.2)
India	9	(7.4)	0	(0.0)
Pakistan	8	(6.6)	2	(22.2)
Libya	3	(2.6)	1	(11.2)*
USA	2	(1.7)	0	(0.0)
Yemen	2	(1.7)	0	(0.0)
Afghanistan	1	(0.8)	0	(0.0)
Germany	1	(0.8)	0	(0.0)
Iraq	1	(0.8)	0	(0.0)
Jordan	1	(0.8)	0	(0.0)
Lebanon	1	(0.8)	0	(0.0)
Malaysia	1	(0.8)	0	(0.0)
Sweden	1	(0.8)	0	(0.0)
Syria	1	(0.8)	0	(0.0)
Turkmenistan	1	(0.8)	0	(0.0)
**Presenting clinical features**†				
Sore throat	95	(78.5)	7	(77.8)
Cough	89	(73.6)	9	(100.0)
Coryza, congested or runny nose	74	(61.2)	7	(77.8)
Enlarged tonsils	65	(53.7)	8	(88.9)
Headache	54	(43.8)	4	(44.4)
Fever (even if subjective)	48	(39.7)	4	(44.4)
Sputum	45	(37.2)	5	(55.6)
Sneezing	38	(31.4)	4	(44.4)
Hoarseness	20	(16.5)	1	(11.1)
Palpable lymph nodes	16	(13.2)	1	(11.1)
Red conjunctiva	13	(10.7)	3	(33.3)

*Mixed infection (*Haemophilus influenzae* and *Moraxella catarrhalis*).

†Clinical features are not independent.

### Clinical features

The majority of enrolled pilgrims complained of sore throat (78.5 %, *n*=95), cough (73.6 %, *n*=89), coryza or congested/runny nose (61.2 %, *n*=74) and enlarged tonsils (53.7 %, *n*=65). However, other clinical features included headache (43.8 %, *n*=54), fever (even if subjective) (39.7 %, *n*=48), sputum production (37.2 %, *n*=45), sneezing (31.4 %, *n*=38), hoarseness (16.5 %, *n*=20), palpable lymph nodes (13.2 %, *n*=16) and red conjunctiva (10.7 %, *n*=13). The demographic and clinical features of the enrolled pilgrim cohort are detailed in Table 1.

### Detection of bacterial agents

Bacterial pathogens were detected in 7.4 % (*n*=9) of the total number of cases. *Haemophilus influenzae *, *Streptococcus pneumoniae* and *Staphylococcus aureus* were detected in 5 % (*n*=6), 1.7 % (*n*=2) and 0.8 % (*n*=1) of the cases, respectively. A mixed infection of *H. influenzae* and *Moraxella catarrhalis* was detected in one case.

All infected cases (*n*=9) included cough in their clinical profile. The majority of infected cases also had enlarged tonsils (88.9 %, *n*=8), sore throat (77.8 %, *n*=7), coryza or congested/runny nose (77.8 %, *n*=7) and sputum production (55.6 %, *n*=5). Other signs or symptoms such as fever (even if subjective) (44.4 %, *n*=4), headache (44.4 %, *n*=4), sneezing (44.4%, *n*=4), red conjunctiva (33.3 %, *n*=3), hoarseness (11.1 %, *n*=1), and palpable lymph nodes (11.1 %, *n*=1) were detected at lower rates among the infected cohort.

### Characterization of AMR phenotypes

All of the *H. influenzae* isolates were resistant to ampicillin (Table 2). In addition to ampicillin, one *H. influenzae* isolate was resistant to trimethoprim/sulfamethoxazole, another to cefuroxime, and one to cefuroxime, cefepime and cefotaxime (Table 2). Additionally, all *H. influenzae* isolates were sensitive to piperacillin/tazobactam and meropenem.

**Table 2.** List of bacteria recovered from patients complaining of upper respiratory tract infections with corresponding antibiotic prescriptions and resistance during the 2018 Hajj

No.	Gender	Age (years)	Antibiotic prescribed	Cultured organism	Resistant to:
1	m	65	None	* Haemophilus influenzae *	Ampicillin and cefuroxime
2	m	57	None	* Haemophilus influenzae *	Ampicillin
3	m	46	Amoxicillin	* Haemophilus influenzae *	Ampicillin and TMP-SMZ
4	f	48	Amoxicillin	* Haemophilus influenzae *	Ampicillin, cefuroxime, cefepime and cefotaxime
5	m	51	Amoxicillin	* Haemophilus influenzae *	Ampicillin
6	m	70	Amoxicillin	* Haemophilus influenzae *	Ampicillin
				* Moraxella catarrhalis *	Ampicillin and TMP-SMZ
7	m	60	Amoxicillin	* Streptococcus pneumoniae *	–*
8	m	51	Amoxicillin	* Streptococcus pneumoniae *	–*
9	m	21	None	* Staphylococcus aureus *	Oxacillin

*Sensitive to the antibiotics used.

TMP-SMZ, trimethoprim/sulfamethoxazole.

*Streptococcus pneumoniae* isolates (*n*=2) were sensitive to a panel of antibiotics including amoxicillin/clavulanic acid, piperacillin/tazobactam, ceftriaxone, cefoxitin, ciprofloxacin, gentamicin and trimethoprim/sulfamethoxazole. The *Staphylococcus aureus* isolate was resistant to oxacillin [methicillin-resistant *S. aureus* (MRSA)] (Table 2). However, this isolate was sensitive to clindamycin, erythromycin, gentamicin, levofloxacin, linezolid, trimethoprim/sulfamethoxazole and vancomycin. The *M. catarrhalis* isolate was resistant to trimethoprim/sulfamethoxazole and ampicillin (Table 2). However, this isolate was sensitive to cefepime, cefuroxime, cefotaxime, meropenem and piperacillin/tazobactam.

### Rate, patterns and appropriateness of antibiotic prescription

Our researchers noted that amoxicillin and azithromycin were the only two antibiotics available in the holy site primary healthcare centres involved in this study. Nearly half of the pilgrims enrolled in this study (52.1 %, *n*=63) had been prescribed and received either amoxicillin or azithromycin (antibiotic prescription rate of 52.1 %). Of these, the majority had received amoxicillin (81 %, *n*=51), while 19 % (*n*=12) received azithromycin.

Most of the pilgrims who received antibiotics (90.5 %, *n*=57 of 63) did not have any detectable bacterial URTI, so antibiotic prescription was not necessary. Moreover, three of the nine patients (33.3 %) with bacterial URTIs did not receive antibiotics (underdiagnosed). The remaining six patients (66.7 %) received ineffective antibiotic treatment (amoxicillin) as the sensitivity tests indicated that they were infected with ampicillin-resistant bacteria. Therefore, the rate of appropriate practices for managing bacterial URTIs in this study during Hajj was 45.5 %, as 55 (out of 121) pilgrims were not infected by bacteria and did not receive antibiotics (Fig. S1).

### Predicting bacterial URTIs through clinical findings

The age range for the nine pilgrims with positive bacterial URTIs was 21–70 years (median 51; IQR 48–60), of which eight were male (8 : 1 male/female ratio). After cross referencing the clinical findings with confirmed bacterial infections, we found that the symptoms most associated with a bacterial infection were cough, enlarged tonsils and sore throat. Although all the bacterial infections were found in pilgrims complaining of cough (9/9, 100 %), most coughing pilgrims were not infected with a detected bacteria (80/89, 90 %). Similarly, the majority of pilgrims complaining of enlarged tonsils and sore throat did not have a bacterial infection (57/65, 87.7 % and 88/95, 92.6 %, respectively), and most of the infected pilgrims complained of both (8/9, 88.9 % and 7/9, 77.8 %, Table 1).

Red conjunctiva was not commonly expressed by pilgrims (*n*=13, 10.7 %) and the sensitivity of its use as a clinical sign for a confirmed bacterial URTI is low (33 %). However, the odds of having a bacterial URTI was found to increase five-fold if a pilgrim presented with red conjunctiva (95 % CI 1.10–23.57), with high specificity (91 %). Conversely, enlarged tonsils had a high sensitivity (89 %) but moderate specificity (50 %). However, pilgrims presenting with enlarged tonsils were eight times more likely to have a bacterial URTI than those without enlarged tonsils (95 % CI 0.97–66.09). Table 3 shows the sensitivity, specificity, PPV, NPV and OR for use of clinical findings to predict a bacterial infection.

**Table 3.** The prediction of some clinical findings to detecting a bacterial upper respiratory tract infection during Hajj

Clinical findings	Sensitivity (%)	Specificity (%)	PPV (%)	NPV (%)	OR	95 % CI
**Gender**						
Male	89	11	7	92	0.96	[0.11–8.35]
Female	11	89	8	93	1.04	[0.12–9.05]
**Age, years**						
0–29	11	86	6	92	0.75	[0.09–6.41]
30–49	22	50	3	89	0.29	[0.06–1.46]
50–79	67	64	13	96	3.60	[0.85–15.18]
**Clinical signs**						
Cough	100	29	1	100	na	na
Enlarged tonsils	89	50	13	98	8.00	[0.97–66.09]
Red conjunctiva	33	91	23	94	5.10	[1.10–23.57]
Coryza, congested or runny nose	78	40	9	96	2.35	[0.47–11.83]
Sputum	56	64	11	95	2.25	[0.57–8.86]
Sneezing	44	70	11	94	1.84	[0.47–7.28]
Palpable lymph nodes	11	87	6	92	0.81	[0.09–6.95]
Hoarseness	11	83	5	92	0.61	[0.07–5.17]
Fever (even if subjective)	44	61	8	93	1.24	[0.32–4.87]
Sore throat	78	21	7	92	0.95	[0.19–4.87]
Headache	44	56	8	93	1.03	[0.26–4.04]

PPV, positive predictive value; NPV, negative predictive value; OR, odds ratio; na, not applicable or cannot divide by zero.

**Fig. 1.**
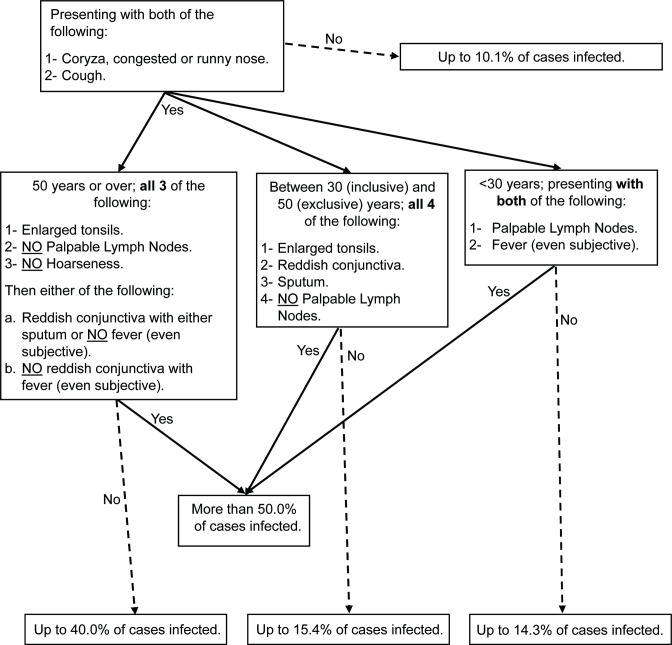
Suggested tool for healthcare workers to predict bacterial upper respiratory tract infections during Hajj. This tool is based on the cumulative effect that the clinical symptom exerts on the probability of a confirmed infection. However, further research on larger sample sizes is required to produce a full guideline.

Pilgrims with more than one URTI clinical finding were more likely to be associated with a bacterial infection. We commonly found that bacterial infections caused the following combination of clinical presentations: nearly all pilgrims who are 29 years or younger presented with coryza, congested or runny nose; fever (even if subjective); palpable lymph nodes; and cough. Moreover, up to 66.6 % of pilgrims aged 30–49 years presented with coryza, congested or runny nose; enlarged tonsils; no palpable lymph nodes; cough with sputum and red conjunctiva had a bacterial infection. For pilgrims who were 50 years or over with enlarged tonsils; no palpable lymph nodes; cough with no hoarseness; and coryza, congested or runny nose, up to 66.6 % were infected if they also had fever (even if subjective) with no red conjunctiva, or nearly all were infected if they had red conjunctiva associated with sputum production, no fever (even if subjective) or both. Fig. 1 summarizes the accumulative effect of the clinical findings to predict a bacterial URTI.

## Discussion

A number of studies have highlighted URTIs and their symptoms as being among the most common complaints of pilgrims and responsible for most healthcare visits. However, very little information is known on the structure of the microbial population and the contribution of AMR bacteria associated with such illnesses in Hajj settings.

*Haemophilus influenzae* was the most commonly isolated bacterial species in our study, accounting for 60.0 % of recovered bacterial isolates (66.7 % of the bacterial infected cohort). Similar findings were seen in Malaysian Hajj pilgrims, with *H. influenzae* accounting for 60.0 % of bacterial isolates (70.9 % of the infected cohort) [35]. Moreover, *H. influenzae* was the most commonly detected pathogen in French pilgrims with respiratory infections for four consecutive Hajj seasons (2014–2017) [15]. Likewise, it is the most commonly isolated organism from patients with respiratory illness within facilities serving Hajj pilgrims [36, 37].

*Streptococcus pneumoniae* in Hajj is usually associated with pneumonia, a lower respiratory tract infection [38]. Moreover, it is the leading cause for respiratory hospital admissions during Hajj [23, 37]. However, URTI studies in Hajj document *Streptococcus pneumoniae* nasal carriage instead of infections [15, 39]. Here the rate of *Streptococcus pneumoniae* infection is low, with only 22.2 % of infected pilgrims detected to be carrying it. No emergence of resistance was seen within the isolated *Streptococcus pneumoniae *.

We found that all *H. influenzae* isolates were resistant to ampicillin. Interestingly, this poses a challenge to the primary healthcare centres during Hajj as they only provide azithromycin and amoxicillin, with *H. influenzae* likely to be resistant to the latter. Furthermore, other prominent bacterial organisms that were isolated in this study (* Staphylococcus aureus *, *Streptococcus pneumoniae* and *Moraxella catarrhalis *) were also resistant to ampicillin. Hence, Hajj health authorities should review and update their antibiotic formulary that is provided to primary healthcare centres in the holy sites and provide suggestions on alternative choices of antibiotics. We suggest that Hajj-deployed physicians refrain from prescribing amoxicillin to pilgrims with suspected URTIs during Hajj.

Clinical decision-making when using antibiotics to treat URTIs is based on the treating healthcare worker’s clinical assessment of the presenting case and their associated medical background knowledge. Some may rely on institutional guidelines or their own personal experience. We found that more than half of the pilgrims (52.1 %) presenting with URTIs in Hajj were prescribed antibiotics, of which 90.5 % were unnecessarily given. The available literature shows a similar prescription rate (47.6 %) of antibiotics for respiratory complaints during Hajj [24]. However, these French pilgrims had a lower rate (60.4 %) of unnecessary prescriptions [24]. The most likely reason for the discrepancy between these two studies is the method for detecting appropriateness. Our study rate was based on confirmed laboratory results within Hajj context. However, the other study values were based on clinical criteria developed for non-Hajj contexts.

We found that only 45.5 % of prescribing practices when managing bacterial URTIs in Hajj were appropriate (from a clinical perspective). However, the previously mentioned study had a higher rate (63.6 %) of appropriate practices [24]. Furthermore, despite the low appropriateness when prescribing antibiotics for URTIs during Hajj, when Hajj healthcare providers refrain from prescribing antibiotics, the decision is 95 % (55 out of 58) appropriate. This is similar to that found in the other study: 86 % (338 out of 395) appropriate [24]. Despite the variation in appropriateness between physicians when prescribing antibiotics for URTI patients in Hajj, they seem to have high accuracy and agreement on those who do not require such a prescription. This observation may be due to the clinical presentations for these pilgrims, which are clearly not those of a bacterial URTI at the time of consultation.

These findings highlight the need to implement an antibiotic treatment guideline that is based on laboratory identification of the aetiologic agents and AMR profiles rather than clinical assessment alone. It is key to first identify the causative agents (viruses and/or bacteria) associated with the URTI and identify the AMR profile of bacterial infections (AMR structure) before any antibiotics are prescribed.

Many pathogens, including bacteria, viruses and fungi, have been identified as the causative agents of URTIs. However, it is challenging to distinguish bacterial infections based on clinical presentation alone. For example, cough, red conjunctiva and coryza are common symptoms associated with URTIs in many studies, including ours. This can be very challenging in Hajj settings where other non-infectious components may be involved. These include environmental (e.g. air pollution and respiratory irritants) or seasonal factors (e.g. viral infections, seasonal changes and allergies) [15, 40–46]. These causes may mimic, interact with or coincide with infectious aetiologies and produce similar clinical findings.

While laboratory confirmation of bacterial URTIs and their associated AMR profile might not be available or practical in Hajj settings, the data provided here have demonstrated some evidence connecting a constellation of clinical presentations to a confirmed bacterial infection. However, further studies including randomized trials with larger sample sizes are required for the development of guidelines to treat URTIs in Hajj settings.

This study has a number of limitations. During Hajj, with the crowding of patients waiting for healthcare services and the limited spaces available, it was challenging to assess the full clinical picture of the pilgrims enrolled in the study. Moreover, many patients were be eager to leave after receiving their medication, and thus some clinical information may have been missed or was not assessed for some pilgrims. Other limitations to our study include the small sample size, male-skewed sample, limited recruitment from Holy site hospitals, no accompanying molecular analyses available and no cost-effectiveness analysis being available.

The most likely explanation for the skewed effect of male participants is that 81 % of our research team were male. Thus, female patients are likely to have been deterred from participating in a study with a male researcher. This highly skewed result is not seen with studies that have a more balanced number of male and female researchers [29, 47–50]. Hence, it is important for researchers intending to undertake Hajj field studies to include a balanced cohort of investigators and co-researchers, to potentially avoid skewed results. Further studies with larger sample sizes representing the diverse pilgrim population may improve our understanding of the dynamics of the transmission of URTIs at Hajj and help in identifying therapeutic and preventive measures.

## Supplementary Data

Supplementary material 1
