# Supplementary material for: Evaluation of the rate, pattern and appropriateness of antibiotic prescription in a cohort of pilgrims suffering from upper respiratory tract infection during the 2018 Hajj season
Source: Access Microbiol. 2022 Apr 25;4(4):000338. doi: 10.1099/acmi.0.000338 (PMC9260093; doi:10.1099/acmi.0.000338)
Supplement: Supplementary material 1 [file acmi-4-0338-s001.pdf]

Supplementary Table 1: the bacterial species identified by Vitek® 2 - compact system (bioMérieux, Marcy-l'Étoile, France) cards GP and GN:

	Identifiable bacterium		
GP (from https://www.biomerieux.com.au/product/vitek-2-gp-id-card)	Abiotrophia defectiva	Leuconostoc mesenteroides ssp. mesenteroides	Staphylococcus xylosus
	Aerococcus urinae	Leuconostoc pseudomesenteroides	Streptococcus agalactiae
	Aerococcus viridans	Listeria grayi*	Streptococcus alactolyticus
	Alloiococcus otitis	Listeria innocua*	Streptococcus anginosus
	Dermacoccus nishinomiyaensis/ Kytococcus sedentarius	Listeria ivanovii*	Streptococcus canis
	Enterococcus avium	Listeria monocytogenes*	Streptococcus constellatus ssp. constellatus
	Enterococcus casseliflavus	Listeria seeligeri*	Streptococcus constellatus ssp. pharyngis
	Enterococcus cecorum	Listeria welshimeri*	Streptococcus cristatus
	Enterococcus columbae	Micrococcus luteus/lylae	Streptococcus downei
	Enterococcus durans	Pediococcus acidilactici	Streptococcus dysgalactiae ssp. dysgalactiae
	Enterococcus faecalis	Pediococcus pentosaceus	Streptococcus dysgalactiae ssp. equisimilis
	Enterococcus faecium	Rothia dentocariosa	Streptococcus equi ssp. equi
	Enterococcus gallinarum	Rothia mucilaginosa	Streptococcus equi ssp. zooepidemicus
	Enterococcus hirae	Staphylococcus arlettae	Streptococcus equinus
	Enterococcus raffinosus	Staphylococcus aureus [a]*	Streptococcus gallolyticus ssp. gallolyticus
	Enterococcus saccharolyticus	Staphylococcus auricularis	Streptococcus gallolyticus ssp. pasteurianus
	Erysipelothrix rhusiopahiae	Staphylococcus capitis	Streptococcus gordonii
	Facklamia hominis	Staphylococcus caprae	Streptococcus hyointestinalis
	Gardnerella vaginalis	Staphylococcus carnosus ssp. carnosus	Streptococcus infantarius ssp. coli (Str. lutetiensis)
	Gemella bergeri	Staphylococcus chromogenes	Streptococcus infantarius ssp. infantarius
	Gemella haemolysans	Staphylococcus cohnii ssp. cohnii	Streptococcus intermedius
	Gemella morbillorum	Staphylococcus cohnii ssp. urealyticus	Streptococcus mitis/Streptococcus oralis
	Gemella sanguinis	Staphylococcus epidermidis*	Streptococcus mutans
	Globicatella sanguinis	Staphylococcus equorum	Streptococcus ovis
	Globicatella sulfidifaciens	Staphylococcus gallinarum	Streptococcus parasanguinis
	Granulicatella adiacens	Staphylococcus haemolyticus	Streptococcus pluranimalium
	Granulicatella elegans	Staphylococcus hominis ssp. hominis	Streptococcus pneumoniae
	Helcococcus kunzii	Staphylococcus hominis ssp. novobiosepticus	Streptococcus porcinus
	Kocuria kristinae	Staphylococcus hyicus*	Streptococcus pseudoporcinus

	Kocuria rhizophila	Staphylococcus intermedius*	Streptococcus pyogenes
	Kocuria rosea	Staphylococcus kloosii	Streptococcus salivarius ssp. salivarius
	Kocuria varians	Staphylococcus lentus	Streptococcus salivarius ssp. thermophilus
	Lactococcus garvieae	Staphylococcus lugdunensis	Streptococcus sanguinis
	Lactococcus lactis ssp. cremoris	Staphylococcus pseudintermedius	Streptococcus sobrinus
	Lactococcus lactis ssp. lactis	Staphylococcus saprophyticus	Streptococcus suis I
	Lactococcus raffinolactis	Staphylococcus schleiferi	Streptococcus suis II
	Leuconostoc citreum	Staphylococcus sciuri	Streptococcus thoraltensis
	Leuconostoc lactis	Staphylococcus simulans	Streptococcus uberis
	Leuconostoc mesenteroides ssp. cremoris	Staphylococcus vitulinus	Streptococcus vestibularis
	Leuconostoc mesenteroides ssp. dextranicum	Staphylococcus warneri	Vagococcus fluvialis
GN (https://www.biomerieux.com.au/product/vitek-2-gn-id-card)	Achromobacter denitrificans	Pseudomonas luteola	Enterobacter gergoviae*
	Achromobacter xylosoxidans	Pseudomonas mendocina	Escherichia coli*
	Acinetobacter baumannii complex	Pseudomonas oleovorans	Escherichia coli O157*
	Acinetobacter haemolyticus	Pseudomonas oryzihabitans	Klebsiella pneumoniae ssp. rhinoscleromatis
	Acinetobacter junii	Pseudomonas putida	Kluyvera ascorbata*
	Acinetobacter lwoffii	Pseudomonas stutzeri	Kluyvera cryocrescens
	Acinetobacter radioresistens	Ralstonia mannitolilytica	Kluyvera intermedia* (formerly known as Enterobacter intermedius)
	Acinetobacter ursingii	Ralstonia pickettii	Leclercia adecarboxylata*
	Actinobacillus ureae	Rhizobium radiobacter	Moellerella wisconsensis*
	Aeromonas hydrophila/Aeromonas caviae	Roseomonas gilardii	Morganella morganii ssp. morganii*
	Aeromonas salmonicida	Shewanella algae	Morganella morganii ssp. sibonii
	Aeromonas sobria	Shewanella putrefaciens	Pantoea agglomerans *
	Aeromonas veronii	Sphingobacterium multivorum	Pantoea spp.
	Alcaligenes faecalis ssp. faecalis	Sphingobacterium spiritivorum	Plesiomonas shigelloides
	Bordetella bronchiseptica	Sphingobacterium thalpophilum	Proteus hauseri
	Bordatella hinzii	Sphingomonas paucimobilis	Proteus mirabilis*
	Bordetella trematum	Stenotrophomonas maltophilia	Proteus penneri*
	Brevundimonas diminuta/vesicularis	Vibrio alginolyticus*	Proteus vulgaris
	Brucella melitensis	Vibrio cholerae*	Providencia alcalifaciens*
	Burkholderia cepacia group [b]	Vibrio fluvialis*	Providencia rettgeri
	Burkholderia gladioli*	Vibrio metschnikovii*	Providencia rustigianii
	Burkholderia mallei	Vibrio mimicus*	Providencia stuartii*
	Burkholderia pseudomallei	Vibrio parahaemolyticus*	Rahnella aquatilis*
	Chromobacterium violaceum	Vibrio vulnificus*	Raoultella ornithinolytica

	Chryseobacterium gleum	Brucella melitensis *	Raoultella planticola
	Chryseobacterium indologenes	Burkholderia mallei *	Roseomonas gilardii
	Comamonas testosteroni	Burkholderia pseudomallei*	Salmonella enterica ssp. arizonae*
	Cupriavidus pauculus	Escherichia coli O157*	Salmonella enterica ssp. diarizonae
	Delftia acidovorans	Francisella tularensis*	Salmonella group*
	Elizabethkingia meningoseptica	Yersinia pestis*	Salmonella ser. Gallinarum*
	Francisella tularensis	Budvicia aquatica	Salmonella ser. Paratyphi A*
	Grimontia hollisae	Buttiauxella agrestis	Salmonella ser. Typhi*
	Mannheimia haemolytica	Cedecea davisae*	Serratia ficaria*
	Methylobacterium spp.	Cedecea lapagei*	Serratia fonticola*
	Moraxella group	Citrobacter amalonaticus*	Serratia liquefaciens group*
	Myroides spp.	Citrobacter braakii*	Serratia marcescens *
	Neisseria animaloris/zoodegmatis	Citrobacter farmeri*	Serratia odorifera *
	Ochrobactrum anthropi	Citrobacter freundii*	Serratia plymuthica *
	Oligella ureolytica	Citrobacter koseri*	Serratia rubidaea*
	Paracoccus yeei	Citrobacter sedlakii	Shigella group*
	Pasteurella aerogenes	Citrobacter youngae*	Shigella sonnei*
	Pasteurella canis	Cronobacter sakazakii group+	Yersinia aldovae
	Pasteurella dagmatis	Edwardsiella hoshinae*	Yersinia enterocolitica/frederiksenii*
	Pasteurella multocida	Edwardsiella tarda*	Yersinia intermedia*
	Pasteurella pneumotropica	Enterobacter aerogenes*	Yersinia kristensenii*
	Pasteurella testudinis	Enterobacter amnigenus 1*	Yersinia pestis
	Photobacterium damselae	Enterobacter amnigenus 2*	Yersinia pseudotuberculosis*
	Pseudomonas aeruginosa*	Enterobacter asburiae*	Yersinia ruckeri*
	Pseudomonas alcaligenes	Enterobacter cancerogenus*	Yokenella regensburgei
	Pseudomonas fluorescens*	Enterobacter cloacae complex [b]	

[a] Staphylococcus aureurs claim contains only the subspecies aureus; [b] Species within this group or complex that are OMA Official Methods of Analysis validated claims are Burkholderia cepacia, Cronobacter sakazakii, and Enterobacter cloacae. * OMA Official Methods of Analysis validated claim.

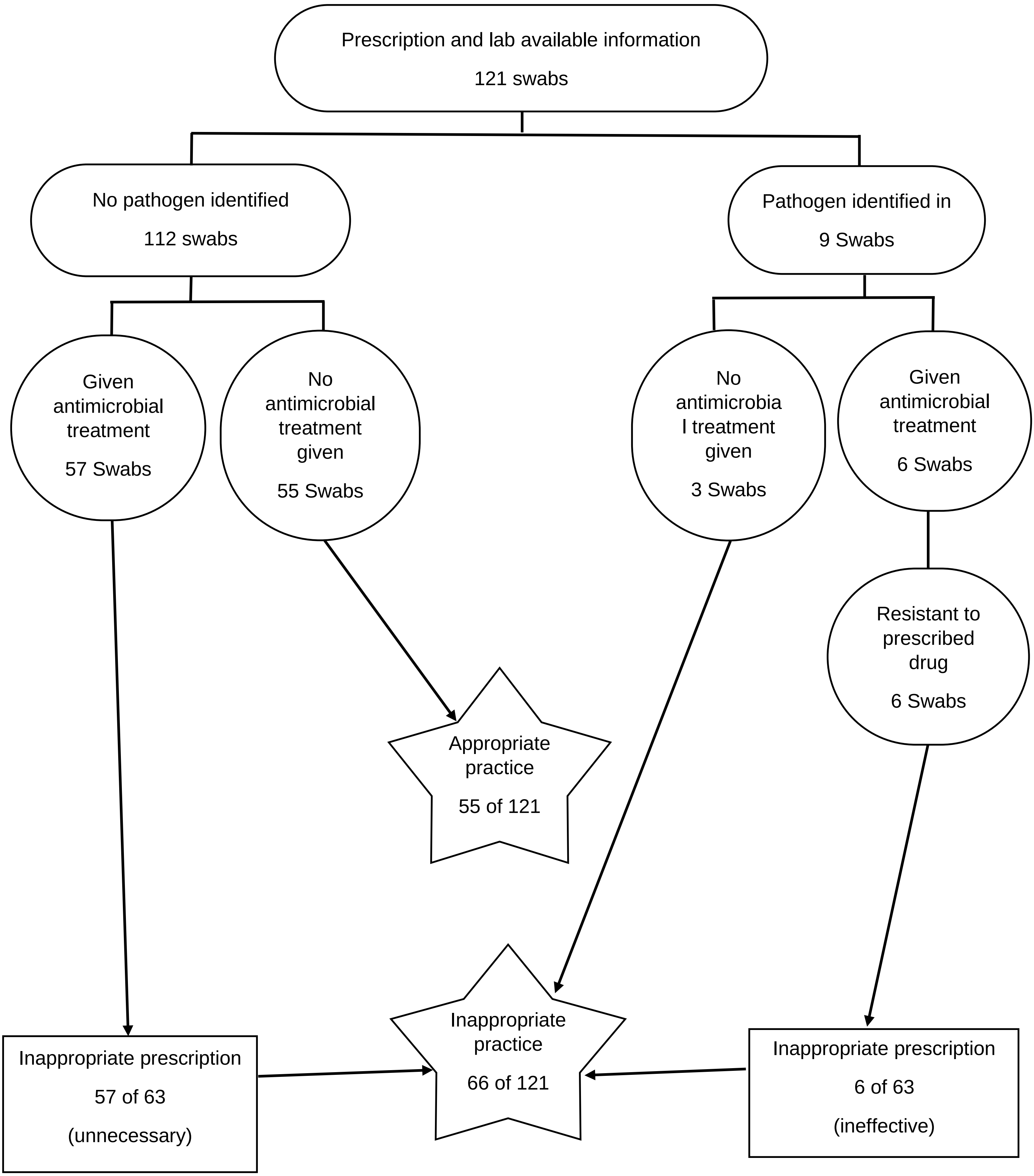

Figure S1: Available information for the appropriateness for treating upper respiratory track infractions during 2018 Hajj season.
